# Beyond Adversity: Definitions, Retrospective Assessment, and Experimental Manipulation of Positive Early-Life Experiences

**DOI:** 10.3390/brainsci16020221

**Published:** 2026-02-12

**Authors:** Erica Berretta, Martina Rizzuti, Laura Petrosini, Francesca Gelfo

**Affiliations:** 1 Department of Human Sciences, Università degli Studi Guglielmo Marconi, 00193 Rome, Italy; e.berretta@unimarconi.it (E.B.); m.rizzuti@unimarconi.it (M.R.); 2 Laboratory of Experimental and Behavioral Neurophysiology, IRCCS Fondazione Santa Lucia, 00143 Rome, Italy; l.petrosini@hsantalucia.it

**Keywords:** adverse childhood experiences, development, early life, modifiable lifestyle factors, neuroplasticity, neuroprotection, positive childhood experiences, enrichment, humans

## Abstract

A wealth of research in neuroscience and developmental psychology has documented the lasting detrimental effects of adverse early-life experiences on health and psychological well-being. To investigate these effects, researchers have developed self- and informant-report questionnaires, interview-based instruments, and experimental paradigms designed to assess exposure to early adversity, model its consequences under controlled laboratory conditions, and investigate the neurobiological mechanisms involved. In contrast, the role of positive early-life experiences in biobehavioral trajectories and adaptive functioning has received comparatively less empirical and theoretical attention. The existing work has largely conceptualized positive experiences in terms of their protective or buffering effects in the context of adversity, and/or their promotive role and independent contribution to physical and psychological well-being. Against this background, this narrative review comprehensively synthesizes (i) current definitions of positive early-life experiences, (ii) tools for their retrospective assessment, and (iii) experimental approaches aimed at manipulating and promoting such experiences in humans. Furthermore, this review advances time-sensitive and individual-centered attention for the study of positive early-life experiences, in which health- and well-being-promoting interventions are informed by an expanding understanding of normative human neuroplasticity as a heterosynchronous process and by dynamic, interdependent interactions operating across individual, family, and societal levels.

## 1. Introduction

Throughout the lifespan, individuals encounter a wide range of experiences. The human brain is wired to detect, respond to, and adapt to environmental inputs, rapidly shaping its structure and function and meeting contextual demands [1,2,3,4]. Neuroplastic processes, including neurogenesis, myelination, pruning, synaptic plasticity, neurotransmitter signaling, and functional reorganization across areas and networks, are particularly pronounced during early development, when heightened sensitivity to experience supports the establishment of fundamental neurobiological functioning and influences later physical and psychological health [5,6,7,8,9,10].

Humans, as an altricial species, are born in a profoundly dependent state. During infancy, survival fundamentally depends on the availability, reliability, and predictability of caregiving figures. The caregiving environment, therefore, constitutes a primary and pivotal source of early-life experiences, operating within a sensitive neurodevelopmental window when the brain is particularly receptive to both beneficial and detrimental signals. These early experiences play a central role in shaping attachment formation, stress regulation, and subsequent cognitive and socio-affective development [11]. When caregiving signals are aberrant, such as when they are inconsistent, unpredictable, or insufficient, early experiences may deviate from species-specific expectancies, potentially shifting the physiological trajectories of structural brain development, neural network organization, and behavioral functioning. Such deviations can have cascading effects across multiple domains of development, increasing vulnerability to later maladaptation [12,13].

### 1.1. Adverse Early-Life Experiences in Developmental Research

The term Adverse Childhood Experiences (ACEs) encompasses several forms of adversity occurring in the early stages of development, including psychological, physical, or sexual abuse; witnessing intimate partner violence; living in adverse or chaotic household conditions; and exposure to caregivers or household members who abuse substances or who are affected by mental illness, incarceration, or suicidality.

The formal consolidation of the ACEs framework is commonly attributed to the epidemiological study by Felitti and colleagues in the late 1990s [14]. Drawing on the data from a large cohort of adult patients, the authors identified a graded association between cumulative exposure to adverse experiences and a wide range of adult health risk behaviors and disease outcomes. Individuals exposed to four or more of the seven categories of ACEs assessed in the study exhibited substantially elevated risks for mental health disorders and maladaptive behaviors, including substance abuse, depression, and suicide attempts. They also showed a higher prevalence of smoking, high-risk sexual behaviors, and poor self-rated health. In addition, cumulative ACEs exposure was associated with an increased risk of several major chronic diseases in adulthood, such as ischemic heart disease, cancer, chronic pulmonary disease, liver disease, and skeletal fractures. The study further demonstrated that different categories of ACEs were strongly interrelated and that individuals exposed to multiple ACEs were significantly more likely to exhibit multiple health risk factors later in life [14].

Collectively, these findings provide compelling evidence that childhood adversity exerts a cumulative and pervasive influence on long-term health, supporting a life-course model in which early psychosocial exposures become biologically and behaviorally embedded over time, contributing to the development of both behavioral and biomedical risk [14,15]. Moreover, the ACEs sequelae can extend beyond the individual, contributing to the intergenerational transmission of risk through biological, psychological, and environmental pathways [16].

Given these outcomes and the substantial individual and societal costs associated with exposure to ACEs, it is not surprising that a significant body of research examines their neurobehavioral consequences in depth and informs the efforts to prevent and mitigate their impact. This sustained scientific interest is driven by clinical urgency and reflects a disease-centric research tradition, embedded within a broader tendency in medicine and psychology to prioritize pathology over well-being [17].

### 1.2. Integrating Positive Early-Life Experiences into Developmental Research

Twenty years after the seminal work by Felitti and colleagues [14], McEwen and Gregerson [18] highlighted important theoretical and methodological limitations of the ACEs framework for investigating developmental trajectories. Traditional ACEs studies often overlooked critical dimensions of the early-life context, including the effects of social and structural inequities. Moreover, the ACEs research has predominantly adopted an impairment-focused perspective, emphasizing risk and pathology while largely neglecting protective factors, supportive relationships, and other positive environmental influences.

The early research on positive early-life experiences was largely grounded in a *resilience*-oriented paradigm that emphasizes the dynamic interplay between protective and risk factors. This perspective emerged from observations of substantial variability in adaptive functioning among individuals considered at risk due to familial, genetic, or socioeconomic disadvantage, or exposure to trauma. The evidence from high-risk populations and case studies demonstrated that, despite comparable adverse exposures, some individuals exhibited more favorable developmental outcomes. These findings suggested the presence of *protective* processes not adequately captured by the models focused exclusively on risk and pathology. Consequently, resilience research sought to identify the factors that support positive adaptation and buffer the effects of adversity, with positive outcomes often conceptualized as the absence of maladaptation rather than the attainment of optimal functioning [19,20].

In the 1970s, *developmental psychopathology* emerged as an integrative, multidisciplinary framework for elucidating the complex interplay between normative developmental processes and the emergence of psychopathology across the lifespan. Grounded in developmental science, this approach posits that maladaptive outcomes cannot be fully understood without considering the dynamic, reciprocal interactions among biological, psychological, and social factors that unfold over time [21,22]. Within this framework, an increasing emphasis has been placed on positive early-life experiences as foundational contributors to later socio-emotional and behavioral development [23].

The emergence of *positive psychology* in the late 1990s provided a further impetus for the systematic investigation of positive early-life experiences. This perspective emphasizes identifying early childhood conditions that promote flourishing, independent of exposure to adversity. In contrast to resilience theory, which focuses on adaptation in the context of risk or adversity, positive psychology is primarily concerned with understanding how positive early-life experiences function as *promotive* factors and with identifying the conditions that foster well-being and successful development, regardless of adverse circumstances [24].

Building on these theoretical foundations, the *Health Outcomes from Positive Experiences* (HOPE) framework offers a conceptual and translational model that explicitly links positive early-life experiences to child health and developmental outcomes across multiple domains, including physical, cognitive, social, and emotional functioning. Rather than focusing solely on risk mitigation, the HOPE framework emphasizes the intentional promotion of positive experiences that support healthy development and well-being, while also simultaneously buffering or preventing the negative effects of adversity [25].

Beyond the differences in theoretical orientation, scholars distinguish between *protective* and *promotive* early-life experiences. *Protective* experiences function as moderators or buffers, reducing the likelihood of adverse outcomes under adversity and increasing the likelihood of positive adaptation [26,27]. In contrast, *promotive* experiences exert their main effects across risk levels, improving outcomes in both low- and high-risk contexts and supporting favorable developmental trajectories regardless of exposure to adversity [26,28,29,30].

Building on the emerging research on positive early-life experiences, the present narrative review is specifically aimed at providing a comprehensive synthesis of the following:

(i) Current definitions;
(ii) Instruments for their retrospective assessment in humans;
(iii) Experimental approaches aimed at manipulating and promoting such experiences in humans.

This review proposes time-sensitive, person-centered attention for the study of positive early-life experiences, emphasizing how interventions aimed at promoting health and well-being can be informed by a growing understanding of normative human neuroplasticity and by departures from typical developmental trajectories. It also highlights the importance of developing and culturally adapting validated tools for the retrospective assessment of positive experiences, with careful consideration of exposure timing and the uniqueness or chronicity of experiences across developmental phases. Finally, this review underscores the need for truly experimental, prospective, and longitudinal approaches to advance the understanding of the effects of both *promoting* and *protective* positive early-life experiences on health and developmental outcomes.

The articles included in this review were identified through searches of the PubMed and Scopus databases, with no temporal restrictions, up to January 2026. Both original empirical studies—including retrospective, cross-sectional, and longitudinal designs—and narrative, systematic, and meta-analytic reviews were considered. Studies conducted in human populations and in animal models were included. Our search strategies involved combinations of the terms “positive experienc*,” “benevolent experienc*,” “resilience,” “development,” “childhood,” “plasticity,” “early-life,” “enrichment,” and related synonyms. This review focuses on the assessment tools and interventions in human populations. The studies employing animal models were included only when they clarified or further elucidated the findings established in humans.

## 2. Definition(s) of Positive Early-Life Experiences

Positive early-life experiences can be operationalized as *promotive* factors that directly reduce the risk of psychopathology and stress and as *protective* factors that buffer or mitigate the impact of adversity, thereby enhancing the likelihood of positive adaptation [26,31]. More broadly, they encompass the experiences children directly encounter, as well as the individual assets and environmental resources that foster connectedness, belonging, resilience, and long-term well-being in typically developing individuals, while also buffering against maladaptation in children exposed to adverse contexts. These experiences can be aggregated into composite indices to quantify their overall exposure or access to protective and promotive factors [32].

The recent literature employs several partially overlapping terms to refer to positive early-life experiences, including Positive Childhood Experiences (PCEs) [28], Benevolent Childhood Experiences (BCEs) [26], advantageous childhood experiences, enriching experiences, and counter-ACEs [33,34].

PCEs, as defined by Bethell et al. [28], include positive parenting, school connectedness, meaningful beliefs, and close relationships with family, friends, and non-parental adults. Using a cumulative PCEs score, the authors assessed its associations with adult mental health across four levels of exposure to ACEs. PCEs were found to frequently co-occur with ACEs and were associated with lower odds of poor mental health and higher levels of social and emotional support reported in adulthood, after accounting for ACEs levels [28]. The results from the cross-sectional study [28] indicate a dose-response association between PCEs and mental and relational health, supporting the conceptualization of positive early-life experiences as *promotive* factors that foster well-being irrespective of adverse circumstances [24]. Notably, three of the seven PCEs considered by Bethell et al. [28] pertain to the caregiving environment. Further empirical evidence suggests that parental warmth in childhood is positively associated with composite measures of flourishing in mid-life (encompassing emotional, psychological, and social well-being) and inversely associated with negative health outcomes [35], reinforcing the *promotive* role of positive early-life experiences.

In the literature, PCEs and BCEs are partially overlapping constructs. However, compared to PCEs [28], BCEs [26,31] focus more specifically on the experiences that buffer children from adversity, emphasizing relationships that mitigate the impact of trauma and risk exposure. Therefore, while the two constructs share common elements, particularly regarding supportive relationships as positive early-life experiences, PCEs highlight their *promotive* role, whereas BCEs emphasize their *protective* role in fostering resilience in high-risk contexts.

When examined as *protective* factors within a resilience-based framework [30], positive experiences such as Safe, Stable, and Nurturing Relationships (SSNRs) during childhood appear to buffer the effects of risk factors, as ACEs, on adverse mental health outcomes. For instance, among individuals exposed to four or more ACEs, those who grew up with a safe and protective adult were significantly less likely to report poor mental health [36]. Moreover, the meta-analytic evidence indicates that SSNRs moderate the intergenerational continuity of child maltreatment, further supporting their *protective* role [37].

Positive early-life experiences extend beyond the family context to encompass multiple system levels. School-related factors, such as the frequency of peer interactions and activities, and perceived school safety, as well as community characteristics, including access to safe play areas and neighborhood safety, are positive early-life experiences that have been identified as important predictors of children’s subjective well-being [38].

From the HOPE framework [25], positive early-life experiences can be organized into four general domains:

(i) The relationship domain, encompassing secure and nurturing connections with family members, adults, and peers;
(ii) The environmental domain, including safe, equitable, and stable environments to live, learn, and play;
(iii) The social domain, reflecting opportunities for social and civic engagement that foster a sense of belonging and connectedness;
(iv) The emotional growth domain.

Among the positive early-life experiences, the research highlights enriching activities, such as playing a musical instrument, taking art or dance lessons, participating in sports, joining scouting or volunteering activities, learning a new language, and going on vacations. These modifiable activities have been proven to improve cognition, health, and well-being across the lifespan and can be leveraged in prevention, intervention, and promotion programs [39,40,41].

Recently, additional enriching experiences have been proposed, including relationships with pets [42] and exposure to nature [43]. The evidence suggests that proximity to and engagement with natural environments can be important childhood experiences that contribute to children’s well-being [43,44]. Exposure to nature during childhood and adolescence, delivered through school-led interventions, has been associated with improvements in mental, physical, and social well-being. Examples of such interventions include outdoor learning activities, nature walks, and the use of green schoolyards, which have demonstrated beneficial effects for children and adolescents aged 5 to 19 years [45,46].

Collectively, a growing body of research from both protective and promotive models converges on the beneficial effects of positive early-life experiences on developmental trajectories and long-term well-being. Positive experiences have been shown to exert favorable effects on physical health, including improvements in cardiovascular fitness [47,48] and associations with reduced risk of chronic health conditions [32,49,50,51]. In addition, positive experiences during childhood predict academic success [52] and enhance cognitive functioning across the lifespan [53,54].

Beyond the cognitive outcomes, PCEs also influence emotional functioning. They are associated with lower levels of emotion dysregulation [55,56] and with greater empathic concern and perspective-taking [57]. Further, PCEs appear to moderate mental health outcomes, contributing to reduced psychopathology [32,49,50,51,58,59] and better prognosis for personality disorders [60,61,62].

## 3. Retrospective Assessment of Positive Early-Life Experiences

The growing interest in positive early-life experiences has prompted the development of instruments for their retrospective assessment. However, in the absence of widely recognized and culturally validated standards, this emerging research has led to the creation of multiple heterogeneous, partially overlapping, and non-exhaustive scores, scales, and measures. The main instruments used in the literature are summarized in Table 1.

*Benevolent Childhood Experiences (BCEs) scale* [26]: A widely used measure is the BCEs scale, a 10-item Yes/No questionnaire designed as a counterpart to the ACEs questionnaire. It assesses positive early-life experiences from birth to age 18 in adults with a history of childhood adversity. In fact, the scale was developed within a developmental psychopathology framework which considers both benevolent and adverse childhood experiences on mental health outcomes.

The BCEs scale evaluates three main domains:

(i) Perceived relational and internal safety and security, such as having at least one safe caregiver and positive core beliefs;
(ii) Positive and predictable quality of life, including enjoyment of school and a stable home routine;
(iii) Interpersonal support, encompassing supportive relationships with teachers, neighbors, extended family, or mentors.

The development of the BCEs scale was grounded in the prior research on Positive Interpersonal Connections (PICs) [63], Protective and Compensatory Experiences (PACEs) [64], and on the dimensions of family-specific strengths identified by Hillis et al. [65], which include family closeness, support, loyalty, protection, love, importance, and responsiveness to health needs.

The psychometric properties of the BCEs scale were initially evaluated in a pilot study involving 101 pregnant women (for details see Table 1) [26]. The results from the sample indicate that ACEs and BCEs are relatively orthogonal and independently contribute to variation in long-term functioning. The predictive validity showed that higher BCEs scores were significantly correlated with lower prenatal depression and post-traumatic-stress-disorder symptoms, perceived stress, and stressful life events. The scale demonstrated excellent test-retest reliability; however, the authors highlighted the potential ceiling effects of the scale, as over one quarter of the sample reported all of the BCEs considered.

Lately, the BCE scale has been further expanded to enhance the dimensional measurement of positive early-life experiences [31]. In fact, the original BCEs included relatively few items addressing aspects of personal identity, the broader community, or other socioecological factors. The 20-item revised version incorporates physical and health-related factors (such as access to nutritious food, adequate medical care, and good sleep quality), public safety factors, and environmental factors (including regular exposure to the outdoors) [31]. The revised version was validated in a sample of 1746 USA young adults, showing good psychometric properties. The BCEs-Revised scores were significantly more strongly inversely associated with all mental health outcomes compared to the original scores. The individuals who identified as one of three racial or ethnic minorities represented in the sample (10.3% Asian, 8.6% Black, 8.4% Latine) were unlikely to report different levels of overall BCEs compared to the individuals who identified as White. The outcome has been taken as promising by the authors, as the expansion of the BCEs items aimed to encompass experiences that could be widely applicable across individuals of varied backgrounds. Nevertheless, the sample only included individuals who were born in and lived in the USA, thus restricting the generalizability of the findings across other groups (for details see Table 1).

*Positive Childhood Experiences (PCEs) score* [28]: The PCEs score is based on a seven-item questionnaire that asks individuals to recall how often or to what extent, during childhood, they:

(i) Felt able to discuss their feelings with family members;
(ii) Felt support from their family during difficulties;
(iii) Enjoyed taking part in community traditions;
(iv) Experienced a sense of belonging in school;
(v) Felt friends’ support;
(vi) Had at least two non-parental adults who showed interest in them;
(vii) Felt safe and protected by an adult in their home [25].

The questionnaire was initially evaluated in a cross-sectional study including 6188 non-institutionalized Wisconsin adults (18 years and older, 50.7% women and 84.9% White). Higher PCEs scores were associated with significantly lower odds of adult depression and/or poor mental health and greater adult-reported social and emotional support, suggesting that PCEs may have lifelong consequences and *promote* mental and relational health despite co-occurring adversities such as ACEs.

The analysis of the PCEs scores was based on a sample from the Wisconsin adult population, which, as stated by the authors, is less diverse than the USA as a whole (which is less diverse than the general population), again hindering the generalizability of results. In addition, PCEs focused on the domain of positive emotional experiences in interpersonal relationships, neglecting other types of positive experiences (such as nature exposure, spiritual experiences, and participation in activities) (for details see Table 1).

Extending the range of positive early-life experiences, the *Health Outcomes from Positive Experiences (HOPE) measure* [66] empirically operationalizes and psychometrically evaluates three primary domains of PCEs, as defined within the HOPE framework:

(i) Nurturing and supportive relationships;
(ii) Safe and protective environments;
(iii) Constructive social engagement and connectedness.

The measure assesses positive early-life experiences from birth to 11 years of age, across four interrelated constructs: (1) positive parenting, (2) trusting and supportive relationships, (3) supportive neighborhood and home learning environments, and (4) social engagement and enjoyment. In a longitudinal study of two cohorts of Australian children, composed of a birth cohort of 5107 infants and a kindergarten cohort of 4983 4-year-olds, a higher exposure to PCEs across these domains has been associated with lower rates of mental health problems and academic difficulties during adolescence [66] (for details see Table 1).

Yet most studies on positive early-life experiences lack standardized protocols for their assessment and frequently rely on self-developed measures [67]. La Charité and colleagues [32] developed a factor-analysis-based PCEs score, consisting of 26 items derived from existing instruments, including the PCEs score [28], the BCEs scale [26], and the HOPE measure [66]. This composite score assesses five domains:

(i) School climate and peer relationships;
(ii) Neighborhood safety;
(iii) Social support;
(iv) Paternal relationships;
(v) Maternal relationships.

Similarly, Slopen and colleagues [47] created a “Positive Childhood Experiences index” by retrospectively assessing eight childhood experiences to examine their association with cardiovascular health in midlife. This index captures multiple dimensions, including parental education, perceived socioeconomic status, two-parent household status, residential stability, absence of smokers in the home, parental warmth, emotional support, and instrumental support [47].

Beyond these measures, some studies have examined enriching experiences before age 13, such as playing musical instruments, learning languages, participating in sports, taking art or dance lessons, scouting, volunteering, and family vacations, which have been associated with improved cognitive outcomes across the lifespan [39].

Moreover, some authors have suggested expanding the traditional retrospective assessments of PCEs and BCEs by incorporating complementary measures of physical activity in nature [68] and general nature exposure [43,69].

Recently, Narayan et al. [70] introduced a scale to measure “childhood centeredness”: emotional connectedness among individuals and their parents or primary caregivers, as well as among household members, from the perspective of the target individual.

Various instruments have been developed to assess positive and adverse childhood experiences across developmental periods simultaneously. The Positive and Adverse Childhood Experiences Survey (PACES) [71] was specifically designed to capture both beneficial and harmful childhood experiences. Its psychometric properties were evaluated in a sample of 728 clients with opioid use disorder. The items loaded cleanly onto two subscales measuring positive versus adverse childhood events and demonstrated the survey’s predictive validity for mental health, drug use, pain, and justice-system involvement [71].

Other instruments use a developmental approach to assess positive and adverse experiences across multiple life stages. For instance, the *Trauma and Attachment Questionnaire* (TAQ) is a 42-item self-report measure that evaluates positive personal experiences, such as competence and safety, alongside negative experiences (including but not limited to neglect, physical, and sexual abuse, and trauma). The TAQ comprises four developmental periods (0–6 years; 7–12 years; 13–18 years; and ≥19 years) across eleven subscales, enabling a comprehensive assessment of the severity and timing of both positive and adverse experiences [72] (Table 1).

**Table 1:** Main measures developed to retrospectively assess positive early-life experiences.

Retrospective Assessment	Domains	Time of Interest	Sample
Benevolent Childhood Experiences (BCEs) scale [26]	perceived relational and internal safety and security; positive and predictable quality of life; interpersonal support	From birth to 18 years of age	101 pregnant women (M = 29.10 years, SD = 6.56, range = 18–44; 37% Latina, 22% African-American, 20% White, 21% biracial/multiracial/other; 37% foreign-born, 26% Spanish-speaking)
BCE-Revised scale [31]	incorporates physical and health-related factors (such as access to nutritious food, adequate medical care, and good sleep quality), public safety factors, and environmental factors (including regular exposure to the outdoors)	From birth to 18 years of age	1746 USA young adults (M = 26.6 years, SD = 4.7, range = 19–35 years; 55.3% female, 42.4% male, 2.3% gender non-conforming; 67.0% White, 10.3% Asian, 8.6% Black, 8.4% Latine, 5.7% other)
Positive Childhood Experiences (PCEs) score [28]	communication and support within the family; community traditions; belonging in school; interpersonal support (friends and non-parental adults)	From birth to 18 years of age	6188 non-institutionalized Wisconsin adults (50.7% women and 84.9% White)
Health Outcomes from Positive Experiences (HOPE) measure [66]	nurturing and supportive relationships; safe and protective environments; constructive social engagement and connectedness	From birth to 11 years of age	2 cohorts of Australian children, composed of a birth cohort of 5107 infants and a kindergarten cohort of 4983 4-year-olds
Positive and Adverse Childhood Experiences Survey (PACES) [71]	positive childhood events; adverse childhood events	From birth to 18 years of age	728 clients at rural Colorado clinics for opioid use disorder treatment
Trauma and Attachment Questionnaire (TAQ) [72]	positive experiences (i.e., competence and safety); negative experiences (i.e., neglect, separation, secrets, emotional abuse, physical abuse, sexual abuse, witnessing, other traumas, and alcohol and drugs)	During four developmental periods: ages 0–6; 7–12; 13–18; ≥19	192 patients with diagnoses of alcohol-related disorders (n = 45), schizophrenic disorders (n = 52), affective disorders (n = 54), and personality disorders (n = 41)

## 4. Experimental Approaches to Positive Early-Life Experiences

Most research on the consequences of ACEs relies on quasi-experimental designs. Early adverse experiences are often assessed retrospectively and cross-sectionally, or the developmental trajectories of individuals already exposed to adverse conditions are followed by using observational longitudinal methods. These approaches have generated valuable insights, although any causal inference remains inherently limited, even when sophisticated statistical or natural-experiment strategies are applied [73].

In this context, animal models of ACEs provide a unique opportunity to experimentally examine the neurobiological mechanisms and effects of adverse early-life experiences in living organisms. Modeling ACEs in animals enables the precise control of adverse exposures; allows the direct testing of hypotheses about the underlying mechanisms at the circuit, cellular, and molecular levels; and facilitates the assessment of the population-level specificity of the observed effects [74,75].

In contrast, positive experiences can be readily implemented and experimentally evaluated in human populations. Their ability to buffer the effects of adversity, enhance well-being, and strengthen psychosocial, cognitive, and neurobiological resources can therefore be rigorously tested under randomized controlled conditions and used to inform the development of intervention, prevention, and promotive programs, also in high-risk contexts.

The research on positive early-life experiences increasingly identifies modifiable lifestyle and environmental factors that can be leveraged to support healthy development and long-term well-being [25]. These factors can be promoted by targeting parenting behavior and implementing evidence-based practices through education, training, or coaching [76]. In parallel, social policies play a critical role in creating supportive contexts by providing and systematically evaluating the impact of home visiting programs, high-quality early childhood education and care, safer neighborhood environments, parental leave policies, and culturally or faith-based community support resources [18,76].

Longitudinal investigations, randomized controlled trials (RCTs), and quasi-experimental intervention studies have identified modifiable resilience factors that can be leveraged to improve health outcomes for traumatized children. These include fostering positive appraisal styles and strengthening executive function, enhancing parenting practices, supporting maternal mental health, promoting parental self-care skills and consistent household routines, and providing anticipatory guidance on the impact of trauma on children [77]. Within a resilience-oriented framework, the strategies to prevent risk and promote protection among children and families focus on reducing or mitigating exposure to adversity (e.g., preventing episodes of homelessness or violence, addressing maternal depression), strengthening access to material and social resources (e.g., housing support, healthcare programs, or financial assistance), and activating protective processes (e.g., enhancing the quality of parent–child relationships, improving parenting practices or foster care arrangements, and facilitating family reunification following disasters) [20].

Among the positive early-life experiences within the familial and social domain, social touch—particularly parental touch—plays a central role during the earliest stages of development [78]. Social touch interventions, defined as intentional, non-invasive tactile practices such as guided caregiver–infant touch and skin-to-skin contact, may constitute positive early-life experiences that promote early learning, strengthen affiliative bonding, and support physiological regulation [79,80,81]. Indeed, gentle skin stroking has been shown to be effective as early as the first weeks of life, with an associated activation of the brain regions linked to primary somatosensory and socio-affective processing [82]. A recent systematic review and multivariate meta-analysis on the physical and mental health effects of touch interventions indicates that these practices are especially effective in promoting weight gain in newborns and reducing pain, depression, and anxiety in both adults and children. Skin-to-skin contact appears to be a critical mediator of these benefits, and familiarity plays a role in newborn outcomes, as parental touch confers greater benefits than clinical staff touch [83,84]. Investigating the impact of touch-based interventions may be particularly relevant for populations at increased risk, including preterm infants, children with neurodevelopmental disabilities, and those exposed to early-life adversity such as neglect [84,85,86].

Early-life enriching experiences encompass a broader range of physical, cognitive, and social stimulations [39,87]. A substantial body of human and animal research has demonstrated the profound effects of multimodal environmental enrichment in promoting cognitive functioning and stress regulation across the lifespan, even in the presence of brain injury and physiological or pathological aging [88,89,90]. Childhood appears to be a particularly suitable developmental period for laying the foundations of the cognitive reserve through enriching environmental experiences [91,92]. The impact of enriching experiences during infancy and childhood may also benefit both young non-clinical and clinical populations. For example, in an RTCs study, sensorimotor enrichment was shown to effectively modulate the severity of autism, as assessed with the Childhood Autism Rating Scale [93].

Physical enrichment, particularly physical activity (PA), is a key experiential stimulus that can be leveraged in targeted interventions. PA has been consistently shown to exert wide-ranging positive effects on brain development, cognitive functioning, socioemotional abilities, and psychological well-being [94]. By stimulating the secretion of serotonin, which is implicated in neuronal proliferation, differentiation, synaptic morphogenesis, and neurotransmission, PA contributes to children’s cognitive functioning and social interaction patterns during early developmental stages [95,96]. Moreover, PA may influence cognitive and emotional outcomes through the epigenetic regulation of neuronal growth and function [97,98,99]. Animal studies have demonstrated marked increases in hippocampal brain-derived neurotrophic factor (BDNF) and mRNA levels in physically active vs. sedentary adolescent mice [100]. In humans, although the evidence is constrained by the reliance on peripheral BDNF measurements, studies support an association between PA and BDNF levels, with the magnitude of this association varying by the type, frequency, and intensity of the physical activity [101].

Based on these findings, promoting PA during childhood is considered particularly important [102]. Progress in the field may benefit from preliminary studies that translate the theoretical principles into structured, evidence-informed PA programs and explicitly address the associated methodological challenges [103]. For instance, pilot RCTs aimed at increasing PA and reducing sedentary behavior among preschoolers in day care settings highlighted several challenges, including the variability in day care provider engagement and the personal investment in the intervention. The authors also reported challenges related to seasonal factors (e.g., children being absent due to vacation periods during the PA protocols or outcome assessments), which may contribute to increased attrition rates [103]. Building on the insights from the pilot studies, the authors proposed potential solutions, such as ensuring that the PA programs are sufficiently straightforward to facilitate compliance and scheduling the outcome assessments outside of vacation periods. Overall, PA represents a key modifiable positive early-life experience, with the preliminary evidence highlighting the need to better delineate sensitive windows for intervention, optimize the type, intensity, and frequency of the PA protocols, and refine the methodological approaches for implementation in familiar, educational, childcare, and leisure settings.

Alongside physical enrichment, socially enriching early-life experiences—ranging from mentoring relationships to organized activities such as arts, music, or scouting, as well as culturally grounded practices—represent valuable and multidimensional sources of positive stimulation.

Notably, some positive early-life experiences, such as exposure to nature and PA, span individual, family, school, and community domains and can be leveraged as low-cost, non-invasive interventions to promote health and psychological well-being [45,46].

Both the retrospective assessments of positive early-life experiences and the intervention studies rely mainly on Western-centric measures, limiting the generalizability of the results. Culturally adaptive research is essential to avoid imposing external definitions of well-being and to ensure interventions that resonate with community values.

## 5. Time-Sensitive and Individual-Centered Approaches to Positive Early-Life Experiences

The research on positive early-life experiences is still in its early stages. Recent reviews and meta-analyses have begun synthesizing the current knowledge on how these experiences affect health and psychological outcomes, both with and without adverse early-life experiences and later-life challenges. The current research offers the opportunity to “administer” positive experiences in a more controlled and ethically feasible manner than ACEs. Therefore, future original studies could systematically investigate positive early-life experiences by differentiating the types of exposure, examining their effects across specific developmental stages, and evaluating their effectiveness in promoting and/or protecting health and well-being in both clinical and non-clinical populations.

### 5.1. Timing of Positive Early-Life Experiences

Most of the retrospective instruments discussed in this review use the term “childhood”. However, La Charité and colleagues [32] assess positive experiences before age 17 for parent-related questions and after age 6 for school- and neighborhood-related questions. The measure, based on the HOPE framework, addresses experiences from birth to age 11 [66], while Morris and coauthors [39] assessed “enriching early-life experiences” before age 13. In contrast, the traditional BCEs [26] and PCEs [28] scales consider experiences from birth to age 18.

A closer examination suggests that these instruments assess experiences that are not confined to childhood but span multiple developmental periods, each characterized by distinct neurobiological features and graded, stage-specific capacities for neuroplasticity.

**Figure 1:**
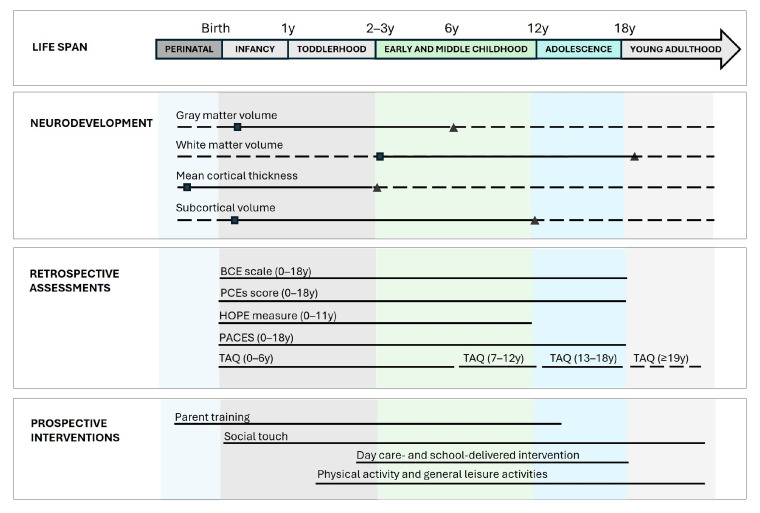
Schematic overview of neurodevelopmental processes (considering [106]) across the lifespan (stages adapted from [104]), highlighting both the age ranges captured by the main retrospective assessment tools for positive early-life experiences and the developmental windows in which prospective interventions can be applied. As in the “neurodevelopment” section, for each measure (i.e., gray matter volume, white matter volume, mean cortical thickness, subcortical volume) squares indicate the peak rate of growth (velocity) and triangles indicate the peak volume (size), derived from human MRI data [106].

Depending on the disciplinary perspective adopted (e.g., medical vs. psychological), the developmental stages may be defined using different age ranges. According to a synthesis by Menezes et al. [104], human development encompasses the neonatal period (from birth to the first four weeks of life); infancy (from four weeks to 1–2 years), often further subdivided into infancy proper (4 weeks to 12 months) and toddlerhood (12 months to 2–3 years); early and middle childhood, including the preschool period (approximately 2–6 years) and the school-age period (6–11/12 years); adolescence (typically spanning 10–18 years and frequently differentiated into early, middle, and late adolescence); and young, middle, and late adulthood. Importantly, the boundaries between the developmental stages vary across disciplines and classification criteria and continue to evolve in response to emerging empirical and theoretical advances, such as the recognition of emerging adulthood as a distinct developmental phase bridging adolescence and full adulthood. Moreover, developmental milestones and the underlying neurobiological processes do not follow discrete, clear-cut trajectories (Figure 1: Life span section).

Human neuroplasticity is neither linear nor uniformly cumulative. Rather, it reflects the dynamic balance between regressive processes, such as synaptic pruning, and progressive processes, including neurogenesis, myelination, dendritic and spine growth, and large-scale functional reorganization of neural networks. These processes unfold through the opening and closing of developmentally sensitive windows and exhibit pronounced heterochronicity across brain regions [105,106]. During infancy, neural development is characterized by exuberant synaptogenesis and rapid circuit formation, whereas later childhood and adolescence are dominated by synaptic pruning, refinement, and specialization of neural networks. Consequently, although the infant brain is highly plastic and particularly responsive to environmental input, the mechanisms that establish foundational circuitry in early life differ fundamentally from those that refine, stabilize, and consolidate mature neural circuits during later developmental stages [107].

Knowledge in this area is rapidly expanding. Large-scale neuroimaging studies spanning development from mid-gestation through older adulthood have used advanced statistical modeling to construct normative human brain growth charts [106]. These investigations reveal nonlinear, region-specific trajectories of brain development across the lifespan, highlighting marked heterochronicity among the neuroanatomical features. For instance, the developmental trajectories differ substantially for gray and white matter volumes, cortical thickness, subcortical structures, and ventricular and cerebellar volumes (Figure 1: Neurodevelopment section). Even within gray matter, the regional changes are heterogeneous and follow a spatially ordered sequence: reductions typically begin in the dorsal parietal cortices, progress rostrally into the frontal cortex, extend caudally and laterally into the parietal and occipital areas, and reach the temporal regions later in development. Notably, the dorsolateral prefrontal cortex maturation is protracted, with volumetric reductions occurring primarily toward the end of adolescence [105,106,108].

Constructing normative brain growth charts enables the definition of typical developmental trajectories across the lifespan. Within this framework, it becomes possible to identify when and how individual trajectories deviate from the normative patterns and to explore the contributions of genetic factors as well as exposure to adverse, positive, or otherwise salient environmental experiences [105,106,108]. Using individualized centile scores or quantile-based mappings situates each individual within age- and sex-specific distributions, providing a standardized metric to characterize neurodevelopmental variability [106]. Moreover, by delineating the periods of accelerated change or heightened neurobiological sensitivity, normative growth charts offer a principled basis for modeling developmentally timed interventions. Such insights may guide hypotheses about the optimal timing of interventions grounded in positive environmental exposures, tailored to the specific neurobiological processes that are most plastic at each developmental stage.

This perspective aligns with the theoretical framework proposed by Tooley et al. [109], which links childhood experiences to brain development across the lifespan. The model holds that experiences earlier in development, particularly educational and cognitively enriching exposures, exert stronger and more enduring influences on neural maturation and plasticity than comparable experiences introduced later in childhood. Rather than producing transient effects, early experiences are proposed to recalibrate the timing and tempo of developmental trajectories, with cascading consequences for later neural and behavioral outcomes. When integrated with normative brain growth charts, the framework underscores that the effectiveness of positive environmental exposures may depend critically on the developmental stage at which they are introduced [109].

Overall, the study of positive early-life experiences should account for advances in our understanding of neuroplasticity trajectories. Currently, there remains a mismatch between the complexity of neuroplasticity across the life stages and the sensitivity of the retrospective tools used to assess positive experiences (Figure 1).

**Figure 2:**
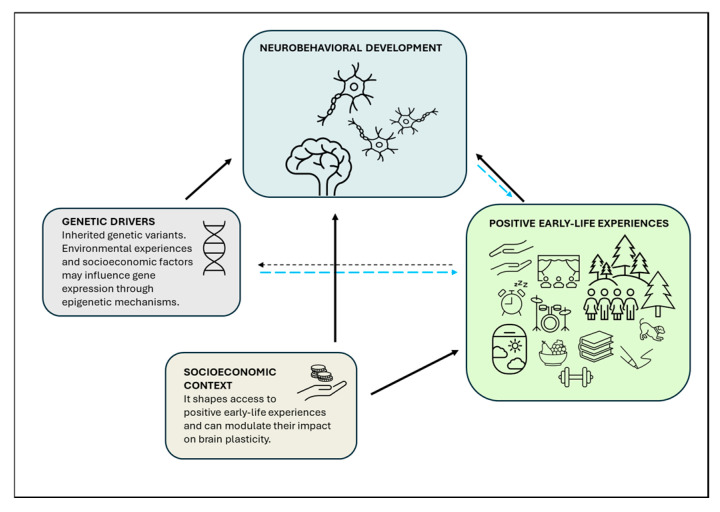
Schematic representation of the influences of positive early-life experiences on neurobehavioral development. Both genetic factors and positive early-life experiences shape neurobehavioral outcomes (black arrows). Individual neurobiological features, such as plasticity levels, can modulate how experiences exert their effects (blue dashed arrow). Genetic and environmental factors also interact through epigenetic mechanisms (black dashed arrow) and gene–environment correlations (blue dashed arrows), whereby genetic predispositions influence the likelihood of encountering certain experiences. Further, socioeconomic status may affect both access to positive early-life experiences and the pace of neurodevelopment (black arrows).

Moreover, socioeconomic variability represents an additional dimension shaping neurodevelopmental trajectories, influencing not only overall well-being, access to positive early-life experiences, and developmental outcomes associated with environmental exposures but also the tempo of brain maturation [109]. The evidence indicates that a higher socioeconomic status during childhood is associated with prolonged structural brain development and a greater segregation of functional networks, thereby adding further complexity to the temporal dynamics of regional brain plasticity (Figure 2).

Chronic exposure to adversity has been linked to accelerated brain maturation, whereas supportive and enriching experiences tend to promote prolonged developmental trajectories, potentially extending periods of plasticity [109]. Adversity may accelerate maturation through sustained activation of stress-regulation circuits, prolonged glucocorticoid secretion, inflammatory processes, and epigenetically mediated cellular aging [109,110]. In contrast, positive environments can prolong and enhance plasticity. In juvenile animals, environmental enrichment increases interleukin-33 (IL-33) signaling, promotes microglial removal of perineuronal nets (PNNs), and elevates neurotransmitters such as noradrenaline, dopamine, and serotonin, thereby supporting synaptic plasticity and cortical development [111,112]. These processes are associated with increased cortical thickness, dendritic branching, spine density, synaptogenesis, and glial proliferation, as well as the regulation of molecular factors, including OTX2 and BDNF, that influence the maturation of parvalbumin-positive interneurons and the timing of sensitive windows [113]. Finally, brake-like mechanisms, such as PNNs and myelin, stabilize neural circuits and close periods of heightened plasticity. Accelerated development, as observed in children exposed to chronic stress or low socioeconomic conditions, may thus shorten windows of heightened plasticity, limiting the brain’s sensitivity to later experiences [109].

### 5.2. Individual-Centered Studies

Positive experiences do not necessarily confer equal benefits on all individuals. Some individuals are more sensitive to environmental influences than others, owing to both genetic differences and prior experiences.

According to the differential susceptibility to environment theory, factors, including personality traits, modulate children’s sensitivity to both adverse and beneficial experiences. For instance, the association between childhood experiences and adult health has been stronger among extroverted individuals than among introverted or ambivert individuals [114], suggesting that individuals with specific personality traits may be more responsive to environmental influences during childhood than others.

Further, genetic variants can influence rates of brain growth [115], thereby modulating how experiences shape brain plasticity and neurobehavioral outcomes. For example, BDNF genotypes moderate the effects of environmental conditions on child neurodevelopment. Specifically, the Val66Met (rs6265) single-nucleotide polymorphism influences attention outcomes in adopted children depending on the duration of institutional care [116,117].

Interventions grounded in positive early-life experiences should account for interindividual differences. For example, in the domain of social touch, the research has shown that the perceived pleasantness of gentle stroking is modulated by personal and contextual factors, including gender, the toucher’s gender, familiarity with the toucher, cultural background, and age. In addition, individual attitudes toward interpersonal touch play a crucial role, such that a pleasant touch experience can turn into disgust depending on the desirability of the toucher [118,119].

The effects of positive early-life experiences, such as social touch, may also vary by clinical condition, with different types of social touch producing distinct outcomes in children with and without neurodevelopmental disabilities [85].

Animal models enable causal investigation of how positive early-life experiences may affect neurobiological mechanisms and how individual differences arising from prior experiences and genetic makeup shape these effects. Methodological advances increasingly enable similar investigations in humans. Recent neuroimaging studies have sought to characterize critical period plasticity during childhood and adolescence [107], often using non-invasive imaging to derive proxy markers of plasticity informed by mechanisms identified in animal models. Rather than measuring plasticity directly, this approach identifies imaging-based signatures whose developmental trajectories and functional properties align with known regulators and consequences of heightened plasticity.

Diffusion magnetic resonance imaging (MRI) has been used to assess the developmental variation in thalamocortical structural connectivity and white matter microstructure. Magnetic resonance spectroscopic imaging enables the in vivo estimation of glutamatergic concentrations, a key component of synaptic plasticity, while contrast and quantitative MRI provide indices of intracortical myelination. Functionally, measures such as the resting-state fluctuation amplitude index of intrinsic neural activity and computational models applied to functional MRI data enable the inference of the excitatory–inhibitory balance by integrating excitatory connectivity, inhibitory signaling, and myelination. Collectively, these imaging-derived measures serve as complementary proxies for developmental plasticity, capturing the changes in circuit maturation, the intrinsic activity, and the balance between evoked and spontaneous neural signals [107]. Combining such methodological advances with controlled true-experimental designs may provide deeper insights into the neurobehavioral impact of interventions promoting positive early-life experiences.

## 6. Conclusions

The growing research interest in positive early-life experiences underscores the need for a more precise theoretical framework, shared operational definitions, refined and validated retrospective measures, and experimental designs to evaluate their impact on health and well-being across the lifespan. Well-designed studies are crucial for informing evidence-based interventions in clinical and primary care settings, including parenting and family programs [99,100], policies that foster community-driven well-being [101], and broader social ecologies that support children in low-resource or humanitarian contexts [102].

Identifying periods of maximal environment-driven plasticity is central to understanding when the developing brain is most vulnerable to disadvantage and, conversely, most responsive to interventions that promote positive experiences.

To date, most neuroimaging studies and validation efforts for retrospective assessment tools have focused on European and North American populations. Culturally adaptive research is therefore essential to avoid imposing external definitions of well-being and to ensure interventions resonate with local community values.

Ultimately, there is no simple intervention for the complex, dynamic trajectories of neurobehavioral development; effective strategies must integrate individual differences, developmental timing, and socioecological context.

## Data Availability

No new data were created or analyzed in this study. Data sharing does not apply to this article.

## Abbreviations

The following abbreviations are used in this manuscript:

ACEs	Adverse Childhood Experiences
ADHD	Attention Deficit/Hyperactivity Disorder
ASD	Autism Spectrum Disorder
BCEs	Benevolent Childhood Experiences
BDNF	Brain-Derived Neurotrophic Factor
HOPE	Health Outcomes from Positive Experiences
LCHD	Life Course Health Development
MRI	Magnetic Resonance Imaging
PA	Physical Activity
PACEs	Protective and Compensatory Experiences
PCEs	Positive Childhood Experiences
PICs	Positive Interpersonal Connections
SSNRs	Safe, Stable, and Nurturing Relationships
TAQ	Trauma and Attachment Questionnaire
RCTs	Randomized Controlled Trials
